# Therapeutics for Graft-*versus*-Host Disease: From Conventional Therapies to Novel Virotherapeutic Strategies

**DOI:** 10.3390/v8030085

**Published:** 2016-03-22

**Authors:** Nancy Y. Villa, Masmudur M. Rahman, Grant McFadden, Christopher R. Cogle

**Affiliations:** 1 Division of Hematology and Oncology, Department of Medicine, University of Florida, Gainesville, FL 32610, USA; nancy.villa@medicine.ufl.edu; 2 Department of Molecular Genetics and Microbiology, University of Florida, Gainesville, FL 32610, USA; masmudur@ufl.edu

**Keywords:** cancer, hematopoietic cell transplant, allogeneic, allo-HSCT, GVHD, GVC, MYXV

## Abstract

Allogeneic hematopoietic stem cell transplantation (allo-HSCT) has a curative potential for many hematologic malignancies and blood diseases. However, the success of allo-HSCT is limited by graft-*versus*-host disease (GVHD), an immunological syndrome that involves inflammation and tissue damage mediated by donor lymphocytes. Despite immune suppression, GVHD is highly incident even after allo-HSCT using human leukocyte antigen (HLA)-matched donors. Therefore, alternative and more effective therapies are needed to prevent or control GVHD while preserving the beneficial graft-*versus*-cancer (GVC) effects against residual disease. Among novel therapeutics for GVHD, oncolytic viruses such as myxoma virus (MYXV) are receiving increased attention due to their dual role in controlling GVHD while preserving or augmenting GVC. This review focuses on the molecular basis of GVHD, as well as state-of-the-art advances in developing novel therapies to prevent or control GVHD while minimizing impact on GVC. Recent literature regarding conventional and the emerging therapies are summarized, with special emphasis on virotherapy to prevent GVHD. Recent advances using preclinical models with oncolytic viruses such as MYXV to ameliorate the deleterious consequences of GVHD, while maintaining or improving the anti-cancer benefits of GVC will be reviewed.

## 1. Introduction

Allogeneic hematopoietic stem cell transplantation (allo-HSCT) involves the transfer of multipotent hematopoietic stem cells from a healthy donor to a genetically non-identical recipient [1]. Allo-HSCT is the most promising therapy used to treat high-risk hematologic malignancies as well as some benign disorders [2]. The major clinical advantage of allo-HSCT is the high degree of anti-cancer effect against residual disease in the transplant recipient following allo-HSCT, also known as graft-*versus*-cancer (GVC) [3], which is mediated by donor T cells and can exert potent anti-neoplastic effects [1]. However, the main clinical complication of allo-HSCT is the development of graft-*versus*-host disease (GVHD), an immunological disorder also mediated by donor T cells. Donor T cells can be very toxic to the recipient, even with close human leukocyte antigen (HLA) matching of the donor and patient. These donor T cells have the potential to attack and damage multiple organs and tissues of the allo-transplanted recipient, resulting in high risk for morbidity and mortality [4].

Despite the progress over the past several years regarding prophylaxis to better control GVHD, the need remains for more effective methods that guarantee not only the control and management of GVHD but also the maintenance, or even improvement, of GVC. Recently, virotherapeutics with oncolytic viruses or genetically modified viruses, have demonstrated potential to promote and improve the GVC effect. For example, myxoma virus (MYXV), a rabbit-specific virus that can mediate oncolysis in a wide spectrum of human cancers [5,6,7,8,9,10,11], has now emerged as a promising *ex vivo* agent to control GVHD and also augment the anti-cancer effects mediated by GVC [12,13].

The pathophysiology of GVHD will be reviewed, focusing on the successes and failures of the associated research and the potential impact of GVHD on allo-HSCT in clinical practice. We also review the current, and most promising emerging therapies to prevent or control GVHD and to elaborate on the strengths and limitations of these strategies. Furthermore, the status of novel therapies to prevent, and control GVHD while preserving GVC will be summarized. Emphasis will be placed on the virotherapeutic strategy with MYXV, which appear to possess the dual consequences of controlling GVHD while at the same time augmenting the anti-cancer effects of GVC.

## 2. Clinical and Biological Overview of Graft-*versus*-Host Disease

### 2.1. Allogeneic Hematopoietic Stem Cell Transplantation Is the Only Potential Cure for Many Blood-Related Diseases, However the Practice Is Limited by Graft-versus-Host Disease

Allo-HSCT is the only curative therapy for many chemotherapy-refractory or relapsed hematologic malignancies [14]. The genesis of allo-HSCT dates back the early 1950s with rodent studies, demonstrating the potential of donated bone marrow to prevent radiation-mediated mortality, which was a great concern with the start of the nuclear age and the Cold War [15,16]. The discovery of human histocompatibility antigens along with the development of immunosuppressive drugs paved the way for the first successful allogeneic bone marrow transplant performed in 1968 [17]. Despite the impact of allo-HSCT, only 25% of patients have a major HLA-identical sibling, whereas a suitable HLA-compatible unrelated volunteer can be located for fewer than 70% of remaining patients [18]. Alternative donors including HLA-haploidentical relatives are now increasingly used [18]. 

#### 2.1.1. Sources of Hematopoietic Stem Cells Influence the Development of GVHD

Several stem cell sources have been used for allo-HSCT. For instance, bone marrow (BM), a rich source of hematopoietic stem and progenitor cells (HSPCs), was the sole source of donor HSPCs until the 1990s (referred to as allogeneic bone marrow transplantation or allo-BMT). In the mid-1990s, donor peripheral blood (PB) that had been mobilized with the cytokines granulocyte colony-stimulating factor (G-CSF) or granulocyte macrophage colony-stimulating factor (GM-CSF) was increasingly used as a source of allo-HSCT [19]. The advantage of using mobilized PB for HSPCT include faster recovery of neutrophil and platelets [20]; however, a drawback of mobilized PB is a higher GVHD risk compared to BM. Umbilical cord blood (UCB) is another source of HSPCs [21]. Whereas UCB transplant is associated with lower risk for GVHD due to higher quantities of more naïve T lymphocytes, the low quantity of hematopoietic stem cells in the UCB unit limits its widespread applicability in engrafting within adults [22,23]. 

#### 2.1.2. Conditioning, Graft Manipulation and Post-Transplant Procedures Affect the Success of Allo-HSCT

Prior to allo-HSCT, patients receive conditioning regimens with chemotherapy and/or radiation in order to (a) kill residual cancer cells in the patient, and (b) inactivate the host immune system so that the allograft will not be rejected. Conditioning is followed by the intravenous infusion of donor HSPCs that seed donor-derived hematopoiesis [24]. In addition, donor-derived immune cells, particularly allo-reactive T lymphocytes, promote immune reactions that control and eradicate residual cancer cells that may have resisted the conditioning chemotherapy and/or radiation. This immunotherapeutic, anti-cancer effect following allo-HSCT is called graft-*versus*-cancer (GVC) and is one of the major therapeutic reasons for allo-HSCT in the treatment of hematological malignancies [25]. Unfortunately, the number of patients eligible for allo-HSCT is limited by the risks and detrimental effects of GVHD, which is a substantial cause of morbidity and mortality after the allo-HSCT. In fact, GVHD occurs in approximately 40%–60% of all allo-transplanted recipients [26] and results in death in up to 20% of these recipients [1,27]. The development and severity of GVHD in transplant recipients depends on recipient age, toxicity of conditioning regimen hematopoietic graft source and the nature of any GVHD prophylaxis.

Another obstacle of allo-HSCT is delayed immune reconstitution, which is exacerbated by the prolonged post-transplant immune suppression required to reduce the risk of GVHD. In this time of immunocompromise, recipients are at very high risk of developing opportunistic diseases like fungal infections, cytomegalovirus (CMV) reactivation, and post-transplant lymphoproliferative lymphomas. It should also be noted that during active GVHD, the patient is also at very high risk of opportunistic infections due to a lack of immunocompetency against microbial infections. Although the prevention and treatment of GVHD induce additional immunocompromise, GVHD itself is an immunopathological condition.

In order to increase the number of patients eligible for allo-transplantation, a balance between ameliorating GVHD and yet preserving GVC is required, as well as the implementation of parameters such as donor selection, stem cell source, conditioning, immune reconstitution, and immunosuppressive regimens [24]. These are the key challenges that need to be addressed in the field of allo-HSCT in order to improve clinical outcomes such as reduced GVHD, elimination of residual disease burden, and increased post-transplant survival times.

### 2.2. Graft-versus-Host Disease: Pathogenesis

Barnes and colleagues initially reported GVHD as a “secondary disease” developed in mice as a result of lethal irradiation and treatment with foreign bone marrow [28]. Later, Billingham defined three essential prerequisites for the development of GVHD: the presence of immunologically competent cells in the donor graft, histocompatibility difference between the donor and the recipient, and the inability of the immunosuppressed recipient to mount an effective immune response to reject the donor cells [1,29]. In 1978, Korngold and Sprent identified mature donor T cells as the fundamental cellular mediators of GVHD [30]. More recently, other factors such as host [31] and donor [32,33] antigen presenting cells (APCs) have also been recognized as crucial in the development GVHD. Today it is clear that the interaction between donor T cells with APCs in general is important for the induction of GVHD. Such interactions can be positively or negatively regulated by a myriad of cytokines, chemokines and other immune cell subsets [34]. Recent studies, however, have demonstrated that donor B cells can also contribute to the immunopathology of GVHD [35,36,37]; but the mechanism(s) by which B cells contribute to GVHD remain elusive.

**Figure 1:**
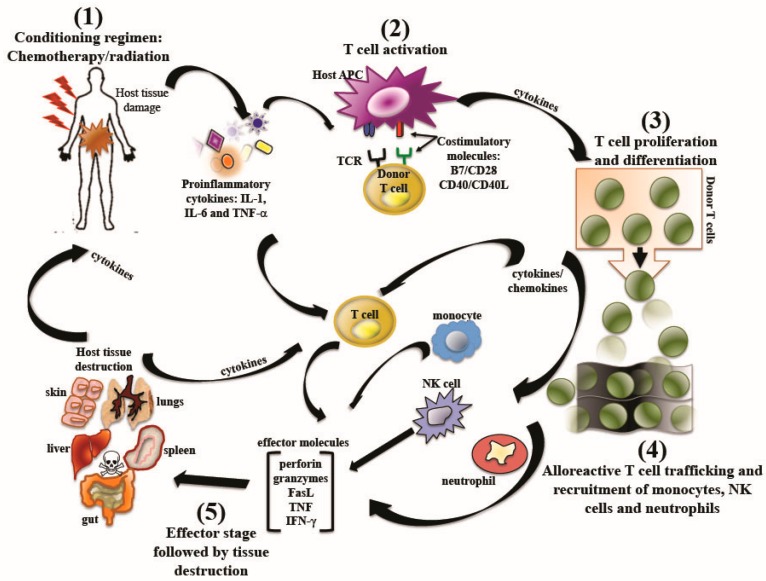
Overview of the pathophysiology of graft-*versus*-host disease. The pathophysiology of graft*-versus*-host disease (GVHD) involves five steps. (1) Conditioning regimen with chemotherapy and/or total body irradiation, which results in the release of pro-inflammatory cytokines that affects host-antigen presenting cells (APCs) by increasing their maturation and the expression of co-stimulatory molecules and cytokines, which in turn fuel donor alloreactive T cells. (2) T cell recognition followed by T cell receptor (TCR) ligation, (the first signal to the donor T cells) and engagement of stimulatory molecules (the second signal to the donor T cells). Both of these signals are required for full T cell activation and subsequent expansion. (3) Proliferation and differentiation of alloreactive donor T cells, followed by (4) trafficking of alloreactive T cells toward GVHD target organs (e.g., skin, gut, liver, lungs), a process that is controlled by chemokines and adhesion molecules. Inflamed and injured tissues produce chemokines, which also result in the recruitment of neutrophils, natural killer (NK) cells, and monocytes to GVHD organs and contribute to the GVHD pathology. (5) The effector phase notes for the destruction of host tissue by effector molecules such as Fas ligand (FasL), perforin, granzymes, interferon-gamma (IFN-γ), tumor necrosis factor (TNF), that induce a cytokine storm that drives the whole GVHD process.

The development of GVHD occurs in five steps, which are schematically represented in Figure 1. The first step involves tissue damage from pre-transplantation conditioning regimens including chemotherapy and/or radiotherapy [20]. The conditioning regimens result in the release inflammatory mediators including tumor necrosis factor (TNF), interleukin-1 (IL-1), adhesion molecules and other danger signals that promote enhancement of the expression of major histocompatibility complex (MHC) proteins [38], the activation and maturation of APCs [39,40], as well as the rapid amplification of donor T cells [41]. The second step corresponds to donor T cell activation and co-stimulation following the recognition and interaction of the T cell receptor (TCR) and the co-stimulatory molecule CD28 with their cognate ligands expressed on the surface of APCs [42]. The third step is the expansion and differentiation of alloreactive T cells into naïve, effector, memory, regulatory, T helper/T cytotoxic cells such Th-1/Tc-1, Th2/Tc2, Th17 and other subsets. Step four is the trafficking of activated T cells to GVHD target tissues, and the further recruitment of other effector leukocytes [38]. Fifth, effector T cells produce toxic cytokines and other immune effectors that induce cell death in the infiltrated recipient tissues. For instance, cytotoxic T lymphocytes, CD4^+^ and CD8^+^, are the major effectors of GVHD and cause tissue damage by a variety of pathways including: the Fas-Fas ligand (FasL) and perforin-granzyme pathways [43], TNF-receptor (TNFR)-like death receptors such as TNF-related apoptosis-inducing ligand (TRAIL) and TNF-like weak inducer of apoptosis (TWEAK) [44,45,46,47,48]. Tissue damage also leads to increased inflammatory signals, perpetuating and augmenting the disease process by contributing to the cytokine proliferation that fuels GVHD [42].

Although APCs and T cells are commonly associated with the development of GVHD, recently, several studies have indicated that non-T cells like neutrophils can also be effectors of GVHD [49,50,51].

Neutrophils promote T cell activation by cleaving chemokines and producing reactive oxygen species (ROS) [52,53]. Thus, in the setting of an allo-HSCT, neutrophils amplify tissue damage caused by conditioning regimens [49]. *In vivo* studies using myeloperoxidase imaging showed that neutrophil infiltration (Ly6G^+^) of mouse ileum after allo-HSCT was dependent of the microbial flora [49]. Neutrophil contribution to GVHD severity occurs via ROS production. Accordingly, deficiency of *Cybb* (encoding cytochrome b-245, beta polypeptide, also known as NOX2) in neutrophils disabled ROS production and led to lower levels of tissue damage, GVHD-related mortality and effector phenotype T cells. In humans, severity of intestinal GVHD correlates with the levels of neutrophils in GVHD lesions [49]. The studies conducted by Schwab and co-workers demonstrated that neutrophils do not contribute directly to GVHD, yet induce tissue damage, which lead to T cell activation and the development of GVHD [49].

Prevention of neutrophil granulocyte infiltration and degranulation can minimize GVHD. 

In this setting, Giroux and co-workers showed that SMAD3 had a role in preventing neutrophils infiltration through the suppression of T helper 1 (Th1) skewing of donor CD4+ T cells.

SMAD3 is a receptor that regulates transforming growth factor-β (TGF-β) signals, [50]. TGF-β signaling plays a key role self-tolerance via the regulation of lymphocyte proliferation differentiation and survival [54]. TGF-β also controls inflammatory responses through the regulation of chemotaxis, activation and survival of lymphocytes, natural killer cells, dendritic cells, macrophages, mast cells and granulocytes [54].

The studies conducted by Giroux *et al.*, demonstrated that the presence of SMAD3 was necessary to decrease the generation of monocytes, neutrophils, as well as the production of ROS by neutrophils. In contrast to wild type (WT), SMAD3- knockout (KO) grafts induced lethal GVHD in major histocompatibility complex-identical recipients. The severity of GVHD was associated with abundant leukocyte infiltration, mainly T cells (CD4^+^) and neutrophils (CD11b^+^Gr-1^+^) into the intestine of recipients [50].

In 2004, a clinical study conducted in humans by Socié *et al.*, demonstrated that increased transplant-related mortality (TRM) was correlated with the expression *in situ* of apoptosis mediators such as TNF-α and Fas in the gastrointestinal (GI) tract during digestive GVHD, as well as increased cellular infiltration of neutrophils in target organs after allo-HSCT [51]. Therefore, a deleterious role of neutrophils on human GVHD adds even more complexity to the pathogenesis of GVHD.

### 2.3. Graft-versus-Host Disease: Clinical Presentations

The clinical presentation of GVHD is heterogeneous, involving the skin, mucosa, GI track, liver and lungs [29]. Based on the time frame and type of pathological process, GVHD can be characterized as acute or chronic. Historically, acute GVHD (aGVHD) occurs within 100 days of HSCT, whereas chronic GVHD (cGVHD) occurs beyond 100 days of the HSCT. However, it is now accepted that clinical features of aGVHD and cGVHD may co-exist and that clinical features of cGVHD can even occur within 100 days after transplant [55]. In terms of mediated factors, it has been thought that aGVHD is driven by Th1-type and Th17-type immune responses, whereas cGVHD is predominantly driven by Th2-type responses. However, recent mouse and human studies have demonstrated that such paradigm is not absolute [20,56,57,58,59,60]. Therefore, a full understanding of the pathophysiology underlying aGVHD and cGVHD is still incomplete. However, it is clear that aGVHD and cGVHD involve distinct pathological processes. For instance, aGVHD has strong inflammatory components whereas cGVHD displays more autoimmune and fibrotic features [1]. There are several risk factors that favor the development of aGVHD or cGVHD. For instance, recipient HLA mismatching and the use of unrelated donors had a greater effect on the risk of aGVHD than on cGVHD. Additionally, total body irradiation was strongly associated with aGVHD. On the other hand, the use of female donors for male recipients correlates more with cGVHD. The use of mobilized blood grafts was associated with cGVHD. Older patients are more susceptible to cGVHD [61]. We next briefly summarize the pathophysiology of aGVHD and cGVHD.

#### 2.3.1. Acute Graft-*versus* Host Disease

The incidence of aGVHD varies with incidence of grade II-IV GVHD at 40% in matched related donor (MRD) transplant to 50% matched unrelated donor (MUD) transplant [2]. Acute GVHD primarily affects the recipient’s skin, GI tract and the liver [34]. The immunobiology of aGVHD is very complex, involving a network of immune interactions where the key players are naïve T cells, host and donor APCs, regulatory T cells (Tregs), among others. The pathophysiology of aGVHD involves three steps: inflammation and tissue damage driven by pre-transplant conditioning regimens, activation and clonal expansion of donor T cells like Th1, accompanied with the secretion of inflammatory cytokines, and release of cellular and inflammatory factors culminating with tissues destruction [62,63,64]. The direct triggers for the induction of aGVHD include: disparities between histocompatibility antigens between recipient and donor, conditioning regimen that the recipient/patient received, and GVHD prevention regimen [34].

##### HLA Disparity

HLA disparity can be at the level of major or minor histocompatibility antigens, which are variable among individuals [65]. The incidence of aGVHD increases with the greater degree of HLA mismatch [66,67]. However, in HLA-matched siblings, mismatches in minor histocompatibility antigens also contribute to GVHD [68,69].

##### Conditioning Regimens

Pre-transplant conditioning regimens trigger the innate immune system with the concomitant induction of aGVHD. Chemo- and radio-therapeutic conditioning regimens performed prior the infusion of BMT donor cells induce the release of pathogen-associated molecular patterns (PAMPs), such as the endotoxin lipopolysacchraride (LPS). The interaction of LPS with toll-like receptors (TLRs) like TLR-4 initiates cellular signaling pathways that activate cytokine secretion [41], such as nuclear factor-κB (NF-κB) [70] and pro-inflammatory cytokines including IL-1, IL-6, TNF, and other IFN family members in a process described as a “cytokine storm” [40,41,71,72].

In aGVHD, the immune response is mounted when APCs sense endogenous molecules that are activated after the conditioning regimen-mediated tissue damage, like damage-associated molecular patterns (DAMPs), which present the MHC or minor histocompatibility antigen disparate protein, and provide secondary co-stimulatory signals via CD28, ICOS, CD40, CD30, 4-IBB and OXO40 molecules, and tertiary (cytokine) signals for activation of mediators of aGVHD such as different subsets of the allo-reactive T cells (mediators of aGVHD). The pivotal role of T cells in aGVHD is supported by the complete abrogation of GVHD following depletion of T cells from the allo-graft, an approach that remains the most effective in preventing aGVHD [1]. However, this strategy also compromises GVC.

As mentioned before, conditioning can induce tissue injury and cell death. Dying cells release adenosine 5′-triphosphate (ATP), which is not only a danger signal that triggers innate immune response pathways, but also is the main endogenous ligand of the P2X7 receptor (P2X7R). Activation of P2X7R is a critical step in the pathogenesis of GVHD [73]. *In vitro* and *in vivo* studies have demonstrated that stimulation of APCs with ATP leads to increase expression of CD80 and CD86 and induce a cascade of pro-inflammatory events such as the phosphorylation of signal transducer and activator of the transcription 1 (STAT1), production of IFN-γ and donor T cell expansion, and the reduction of regulatory T cell numbers. Therefore, ATP neutralization, blockage or genetic deficiency of P2X7R during the development of GVHD can improve survival [73].

Furthermore, the interaction between ATP and P2X7R promotes assembly and activation of the Nod-like receptor protein 3 (Nlrp3) inflammasome, an intracellular multiprotein complex that controls the activation of inflammatory caspase-1 in response to exogenous and endogenous stress or danger signals [74]. After conditioning regimen, intestinal commensal bacteria and the damage-associated molecular pattern uric acid contribute to Nlrp3 inflammasome-mediated IL-1β production, which affects GVHD in the early phase after allo-HCT. Thus, gastrointestinal decontamination and depletion of uric acid are alternatives to reduce the severity of GVHD. Moreover, early blockade or genetic deficiency of IL-1β receptor in dendritic cells (DCs) and T cells prolongs survival [75]. Nlrp3 and the apoptosis-associated spec-like protein ASC, two components of the Nlrp3 inflammasome mediate the cleavage of the precursor IL-1 β protein (pro-IL-1 β) into its active form. Accordingly, Nlrp3 and ASC are critical for the full manifestation of GVHD. Increase levels of active caspase-1 and IL-1β have been found in circulating leukocyte and in intestinal GVHD lesions of patients compared with patients without GVHD [75].

Co-stimulation signals are required to achieve T cell activation, proliferation, differentiation and survival [76]. *In vivo* studies have demonstrated that inhibition of co-stimulatory molecules reduces acute GVHD [34,77,78,79]. On the other hand, blockade of inhibitory signals, (for instance, those that inhibit the T cell response) such as programmed death-1 (PD-1) and cytotoxic T lymphocyte associated protein 4 (CTLA-4), exacerbates aGVHD in murine models [80].

As mentioned above, alloreactive donor T cells are the critical mediators of aGVHD. Alloreactive donor T cells consist of several subsets with different stimuli responsiveness, activation thresholds, and effector functions [34]. The composition of host allo-antigens dictates which donor lymphocyte subsets will differentiate and proliferate [4]. Mediators of aGVHD and their implications on the pathophysiology of aGVHD are summarized in Table 1.

#### 2.3.2. Chronic Graft-versus-Host Disease

cGVHD affects 30%–80% of patients receiving allo-HSCT [64] with a 5-year mortality rate of 30%–50%, mainly due to immune regulation and opportunistic infections [1]. cGVHD is a multi-organ syndrome, with a clinical presentation resembling autoimmune vascular diseases, such as systemic lupus erythematosus, Sjögren’s syndrome, lichen planus, and scleroderma [97]. The first symptoms of cGVHD tend can appear around 100 days after the allo-HSCT; however, cGVHD often presents clinically many months to years after transplant [61].

Classically, Th2 responses have been involved in the pathophysiology of cGVHD. However, we now recognize that the biology of cGVHD is much more complex and thus other factors such as thymic dysfunction, transforming growth factor-β (TGF-β) and platelet-derived growth factor (PDGF), B cells and autoantibodies, and Th1/Th2/Th17 cytokines, as well as regulatory T cells (Tregs), need to be taken into consideration [98].

The treatment of cGVHD is based on its severity. For instance, mild cGVHD is treated with topical immunosuppressive agents or systemic steroids. Moderate to severe cGVHD requires treatment with systemic immunosuppression including prednisone with continued administration of calcineurin inhibitor for steroid sparing [99]. A major complication of cGVHD is a steroid-refractory state, which portends for a poor prognosis. Preclinical studies using mouse models demonstrated that the synthetic retinoid Am80 attenuated cGVHD by downregulating Th1 and Th17 differentiation in donor T cells [97,98,100]. More detailed research on the pathophysiology of cGVHD may help establish novel strategies for its prevention and treatment.

## 3. Prevention of Graft-*versus*-Host Disease: Current Clinical Principles and Strategies

Despite the progress in understanding the pathophysiology of GVHD, the limited success of established therapies for prevention and treatment of GVHD remains unsatisfactory. The ideal clinical scenario for allo-HSCT would be prevention of GVHD while retaining GVC. However, it has not been easy to de-segregate GVHD from GVC, since the main effectors for both GVHD and GVC are T lymphocytes that share many common pathways for these two outcomes. 

T lymphocytes contained in the donor graft have crucial roles in the pathogenesis of GVHD; therefore, prophylaxis of GVHD has been focused on the depletion, tolerization or functional incapacitation of donor T cells [1]. The strategies used for the prophylaxis of GVHD rely on the nature of conditioning regimen, donor type, stem cell source and the degree of HLA mismatch [101,102]. Single pharmacological agents or combination of them have been used in an effort to prevent GVHD after allo-HSCT. Other strategies such as the use of monoclonal antibodies and depletion of T lymphocytes have been used actively in the prophylaxis of GVHD.

Various methods have been tested to manipulate donor T cells to retain therapeutic effector functions against residual tumor cells in the patient without causing GVHD. In this section, current strategies that are applied in the clinic for the prevention and treatment of GVHD, are reviewed. In addition, emerging strategies and their potential to improve patient outcomes are discussed.

### 3.1. Limiting Organ Toxicity by Reducing Intensity of Chemotherapy Conditioning

A myelosuppressive regimen is administered prior to allo-HSCT to deplete stem cells from the host, thus making more available engraftment space for the donor graft, and to reduce graft rejection mediated by host immune cells [1]. However, the toxicity and the suppressive effects of this regimen usually result in host inflammation and tissue damage that ultimately accelerate the development of GVHD [1]. As an alternative, reduced intensity conditioning regimens using non-myeloablative stem cells transplantation (NM-SCT) therapies have contributed to improve prophylaxis of aGVHD [103,104]. However, reduced-intensity conditioning regimens rely on the beneficial GVC effects of the donor transplant to eliminate residual disease.

### 3.2. Pharmacological Agents Used in Conventional Graft-versus-Host Therapies: Advantages and Drawbacks

This section summarizes some of the most relevant drugs and therapeutic strategies used for the inhibition or depletion of allo-reactive T cells.

#### 3.2.1 Inhibition of Allo-Reactive T cells

##### Methotrexate (MTX)

MTX inhibits dividing allo-reactive T-cells [105] resulting in protection against GVHD. However, a major limitation of MTX is the observation of aGVHD between HLA-matched siblings [106].

##### Calcineurin Inhibitors

Calcineurin inhibitors such as cyclosporine A (CYA) [107], and tacrolimus (FK506) [108] immunosuppress T lymphocytes [109]. These inhibitors prevent GVHD by inhibiting the activation and proliferation of donor T cells via blocking nuclear factor of activated T cells (NFAT) [107]. CYA also attenuates the expression of IL-2 and IL-2 receptor (IL-2R) in activated T lymphocytes. However, only a mild to moderate prevention of GVHD has been reported in the presence of calcineurin inhibitors.

##### Corticosteroids

Corticosteroids at high doses are the first-line treatment for established GVHD [110]; however, a significant role in GVHD prevention has not been established. For instance, trials using prednisone in combination with cyclosporine and/or methotrexate have not demonstrated improved GVHD prophylaxis [107]. On the contrary, studies using cyclosporine and prednisone has been associated with increased risk of cGVHD [111]. 

##### Cyclophosphamide 

Cyclophosphamide has been used post-transplant since the 1980s for the prevention of aGVHD, which occurs via inhibiting T cell division and is used after myeloablative conditioning in related and unrelated allo-transplants [112].

##### Mycophenolate Mofetil (MMF)

MMF has a potent cytostatic effect on T and B lymphocytes [113]. MMF has been used in combination with calcineurin inhibitor and/or methotrexate. However, the incidence of grade II-IV GVHD still ranged between 38% and 62 [114,115]. Combination of MMF with cyclosporine resulted in faster engraftment, yet the incidence of aGVHD and cGVHD was totally prevented [113].

##### Sirolimus 

Sirolimus (rapamycin) forms a complex with the mammalian target of rapamycin (mTOR), inhibiting biochemical pathways with the concomitant reduction of DNA transcription/translation of protein synthesis, cell cycle progression, which ultimately result in T cell immunosuppression [113]. Sirolimus, in combination with tacrolimus, has been used with good efficacy against aGVHD. However, no effect was observed against cGVHD. In general, the prevention of GVHD with sirolimus has been mild to moderate.

#### 3.2.2. Depletion of T lymphocytes 

T cell depletion prophylaxis for GVHD was undertaken in the 1980s, and 1990s. The three main depletion strategies were *ex vivo* negative selection of T cells, *ex vivo* positive selection of CD34^+^ stem/progenitor cells, and use of antibodies against T cells *in vivo*.

In the 1970s, GVHD was lethal in recipients of HLA-mismatched bone marrow [116]. In the 1980s this obstacle was overcome by removing T cells from the allo-graft, resulting in reduced incidence and severity of GVHD [64,109,117]. Differential agglutination with soybean agglutinin (SBA) was initially performed to deplete T cells in murine models [118], and then successfully translated to patients with severe combined immunodeficiency (SCID) using BSA followed by E-rosette depletion with sheep red blood cells [119,120]. Even in absence of GVHD prophylaxis, no signs of GVHD were detected [64,109,117,121]. Unfortunately, this technique of T cell depletion also led to poor hematopoietic engraftment, increased incidence of disease relapse and increased incidence of opportunistic infections [109,122,123]. Using a magnetic bead approach to deplete T cells from G-CSF mobilized peripheral blood stem cells, allo-HSCT recipients developed minimal GVHD and high rate of engraftment [124]. Allograft depletion of CD3/CD19 with anti-CD3/CD19 coated microbeads not only preserved the levels of CD34^+^ stem/progenitor cells, but also NK cells and dendritic cells [125]. Depletion of alpha/beta T lymphocyte subsets, which are responsible for the occurrence of GVHD [126], was another graft manipulation strategy that was effective for allo-HSCT [126].Polyclonal Antibodies: *In vivo* administration of the polyclonal anti-thymocyte globulin (ATG) antibodies, pre- and peri-allo-HSCT, simultaneously targeted donor and host T cells resulting in the control of GVHD and avoided graft rejection. However, one of the challenges was that the ATG antibodies also target B cells, NK cells and APCs, without reducing the incidence of cGVHD [113].

##### Monoclonal Antibodies (MAbs)

MAbs like T10B9, which targets the T-cell receptor, in combination with CYA have been used to minimize graft rejection; however no improvement in long-term survival has been observed. On the contrary disease relapse, and the risk of opportunistic infections were increased [127]. Alemtuzumab (Campath), is a MAb that reduces the risk of GVHD [113]. Alemtuzumab targets the CD52 antigen, which is expressed in the surface of B and T lymphocytes [113]. One advantage of this antibody is that engraftment is feasible and reduces the non-relapsed mortality after related and un-related transplants [128]. Although administration before allo-HSCT, with either related or un-related donors, resulted in a lower incidence of GVHD, alemtuzumab remains in the blood for up to 1–2 months after transplantation resulting in a delay of immune reconstitution with a higher incidence of viral infection and disease relapse [129]. In an attempt to circumvent these obstacles, selective depletion of T cell subsets including CD4^+^, CD6^+^ and CD8^+^ have been implemented. However, the success of this strategy remains limited [4,109,130,131].

##### Positive selection of CD34^+^

CD34^+^ with immune-magnetic beads is an effective method of depleting allo-reactive donor T cells prior to transplant, and results in significant reduction of aGVHD and cGVHD [132,133]. However, major limitations of this method are increased risk of infections, which results in 40% mortality, and a high incidence of cancer recurrence (51% of patients transplanted) [132,134].

## 4. Treatment of Established Graft-*versus*-Host-Disease

There is no specific Food and Drug Administration (FDA)-approved treatment for established aGVHD or cGVHD. Currently, the first-line therapy for GVHD is with corticosteroids like prednisone [102]. Corticosteroids suppress the immune response by inhibiting the production of inflammatory cytokines and therefore, reducing inflammation. However, the steroid toxicities and their limited responses are frequent challenges of this therapy. In addition, outcomes for patients with corticosteroid-refractory GVHD (SR-GVHD) are dismal with only 5–30% long-term survival [135].

Adjuvant topical steroids to reduce the steroid toxicities have been shown to have some success. In a study evaluating oral beclomethasone, this steroid improved outcomes in patients with GI aGVHD [136]. In another study, clobetasol or dexamethasone applied topically reduced the symptoms of oral cGVHD [137].

A number of approaches to reduce the symptoms of GVHD that include the use corticosteroids in combination with other agents have been tested. These other agents have included horse rabbit anti-thymocyte globulin ATG; daclizumab, a monoclonal antibody that targets CD25 (IL-2Rα) present on activated T lymphocytes, antibodies against TNF (e.g., etanercept or infliximab), pentostatin, MMF, and sirolimus. Unfortunately, these pharmacological agents administered with corticosteroids failed to improve response rates [135]. and are frequently associated with higher non-relapse mortality [138].

A second line of treatment of GVHD includes the use of anti-metabolites (e.g., mycophenolate mofetil or methotrexare), extracorporeal photopheresis or light therapy, which improve GVHD outcomes in skin, liver, and mouth tissue. Photopheresis is complicated and requires up to six months to see improvement of the GVHD symptoms.

For cGVHD treatment, prolonged administration of corticosteroids in combination with calcineurin inhibitors such as cyclosporine or tacrolimus has been used as a typical standard therapy. However this combination therapy has not improved patient outcomes [113].

Although some of the above mentioned therapies have been used a second-line therapy in the treatment of SR-GVHD, none have been adopted as a standard salvage therapy for either SR-aGVHD or SR-cGVHD. Pre-clinical evidence has indicated that ruxolitinib, an inhibitor of the Janus kinase (JAK) 1 and 2, has potent anti-inflammatory properties [139].

Since activated JAKs are required for T-effector cell responses in different inflammatory diseases, their blockade could reduce GVHD [140]. Using a mouse aGVHD model, Spoerl *et al.*, showed that inhibition of the JAK1/2 signaling with ruxolitinib resulted in reduced proliferation of effector T cells, suppression of production of proinflammatory cytokines in response to mouse allo-antigen, and increase in Foxp3^+^ regulatory T cells [140]. Therefore, ruxolitinib may be a promising new therapy for the treatment of SR-GVHD. Recently a retrospective survey involving institutions in United States and Europe, showed high response rates (>80%) and 6-month survival rates in patients with SR-aGVHD or SR-cGVHD [139].

Bortezomib, a proteasome inhibitor, was approved by the FDA for treating patients with multiple myeloma and mantle cell lymphoma [141]. Although bortezomib prevents aGVHD in mice, the inhibitor does treat ongoing aGVHD. Recently, bortezomib was evaluated in a sclerodermatous cGVHD mouse model and as a result cutaneous lesions were improved, along with a reduction of germinal center B cells were observed [142]. B cell dysregulation and allo-antigen production are known to play a critical role in the pathogenesis of cGVHD [143]. In addition to this, bortezomib has an inhibitory effect on B cells and plasma cells [144]. Based on the murine results, a pilot human clinical trial with bortezomib for the treatment of cutaneous cGVHD resulted in positive clinical responses with minimal toxicity demonstrating that bortezomib may be a treatment option for patients with SR-cGVHD [142].

## 5. Alternative and Novel Strategies to Inhibit Graft-*versus*-Host Disease

### 5.1. Attenuating Graft-versus-Host Disease by B Cell Depletion

B cells contribute to immune responses through the production of antibodies, inflammatory cytokines, antigen recognition and other immunoregulatory functions. A growing body of research indicates that in addition to T cells, B cells play an important role in the immune pathology and tissue damage characteristic of GVHD [145]. Thus, the understanding of how B cells contribute to the development of GVHD after allo-HSCT is becoming an active area of investigation [146]. In the setting of allo-HSCT, B-cell-depleting therapy with rituximab has been investigated for the treatment cGVHD [37,145]. Moreover, administration of rituximab prior to allo-HSCT has resulted in reduced incidence and severity of aGVHD [147,148]. Rituximab binds to CD20 on B-lymphocytes and malignant lymphoma causing cell destruction via a multifactorial mechanism that includes antibody-dependent cell mediated cytotoxicity, cell lysis, and induction of apoptosis of target cells. Thus, rituximab leads to the depletion of B cells. Furthermore, an increase in the suppressive function of Tregs has been observed after the treatment with rituximab [149].

### 5.2. Cellular Suicide Gene Therapy

The cellular suicide gene approach is a promising tool to manipulate donor T cell effector functions in order to augment the GVC effects, promote immune reconstitution, and to prevent or control GVHD [134]. The cellular suicide gene approach is based on the transfer of a suicide gene into donor lymphocytes, which allow safe infusion of viable active T cells, which can be selectively controlled post-transplant in the event of GVHD. The herpes simplex virus thymidine kinase (HSV-TK) has been widely used as a suicide gene in humans [134]. The HSV-TK is a cell cycle-dependent suicide gene that catalyzes the generation of triphosphate ganciclovir (GCV). GCV inhibits DNA chain elongation, which is toxic for proliferating cells [150,151]. *In vitro* and *in vivo* preclinical studies and phase I/II clinical trials have demonstrated that the retroviral-mediated transfer of the suicide gene HSV-TK into donor T cells prior to infusion allows for the efficient control of donor T cell allo-reactivity [152]. Importantly, donor suicide gene-modified T cells (SGMTCs) provide beneficial anti-leukemic, anti-viral and immune reconstitution-facilitating effects to recipients of an allo-HSCT [153]. Currently, HSV-TK therapy for GVHD is being evaluated in a phase III clinical trial for high-risk acute leukemia [134].

There are some limitations of the HSV-TK approach. In immunocompromised patients, TK may lead to undesired elimination of transduced cell populations as a result of the immunogenicity of this viral protein. In addition GCV is a drug used to treat cytomegalovirus (CMV) reactivation, which commonly affects immunocompromized patients. Administration of GCV in these patients to treat CMV induces undesired TK-cell killing [134].

A different suicide gene strategy has been explored using inducible human caspase 9 transgene (iC9), which is a hybrid protein consisting of a human FK 506-binding protein (FKBP12) linked to a modified human caspase 9 lacking the caspase recruitment domain (CARD). The iC9 transgene can be dimerized and activated by administration of a bio-inert small molecule drug AP1903. A phase I/II clinical trial suggests that patients had immediate and sustained protection from major pathogens in the absence of aGVHD or cGVHD [154].

### 5.3. Regulatory T Cells

Naturally occurring Tregs are immunophenotypically defined by CD4^+^CD25^+^Foxp3^+^. Tregs constitute 5%–10% of peripheral CD4^+^ T cells, and play a pivotal role in the induction of immunologic tolerance and maintenance of immune homeostasis [155,156]. Additionally to inhibit graft rejection of MHC-disparate allografts after sub-lethal conditioning in mice [123,157,158], adoptive transfer of Tregs can prevent GVHD [94,156,159]. However, the low frequency of Tregs [155,160] represents a major obstacle for the clinical application of Tregs for the prevention or treatment of GVHD. This obstacle has been partially overcome by using methods such as *in vitro* expansion of Tregs through T cell receptor (TCR)-mediated activation with IL-2 [156], *in vitro* conversion of conventional T cells (Tcon) into Tregs in the presence of TGF-β [161], or via gene transfer of Foxp3, a forkhead-box transcription factor, which is defective in autoimmune and inflammatory syndromes [162]. Nevertheless, the major problem is that none of these methods can produce a homogeneous and stable population of cells with a suppressive capacity equal to that of natural Tregs [163].

In 2010, Cao and co-workers [155] reported the use of a lentivirus-based strategy to express Foxp3 ectopically in mouse CD4^+^CD25^−^ T cells. Using a leukemic model, they demonstrated that the infusion of engineered Tregs in combination with donor bone marrow and splenocytes not only prevented recipients from lethal GVHD, but also the GVC effect was maximally preserved [164]. Although the therapeutic potential of engineered Tregs for the separation of GVHD and the GVC effect have been demonstrated, the determination of the kinetics of the lentiviral transduction and the determination of the potential biosafety issues of lentiviral are still pending before this methods can be applied in clinical trials.

### 5.4. Targeting Co-Stimulatory Molecules

Co-stimulatory molecules provide the second signal that fully activates alloreactive T cells. The co-stimulatory pathways are grouped in two major families: the immunoglobulin (Ig) superfamily and the TNF/TNF receptor (TNFR) family. Since co-stimulatory molecules promote GVHD, different therapeutic approaches have been aimed to target and block such molecules. Herein, we briefly describe some of the best-known co-stimulatory molecules and their action of blockade with pharmacological agents to abort GVHD.

#### 5.4.1. CD137

CD137 is a member of the TNF receptor family that functions as a co-stimulatory molecule for T cells. CD137 ligation on CD8^+^ T cells increases T cell survival proliferation and cytotoxic T lymphocyte (CTL) activities. The use of anti-CD137L mAb inhibits aGVHD, while exacerbating cGVHD. The anti-CD137L mAb inhibits the activity of donor T cells in the following GVHD mouse models: DBA/2 (donor)→F1 (host); and C57BL/6 (donor)→F1 (host) [165].

#### 5.4.2. CD28

CD28 is constitutively expressed on naive T cells. CD28 has two ligands, B7-1 and B7-2, which are expressed on APCs; and the activation of T cells induces the up-regulation of these ligands. B7-1 and B7-2 also have high-affinity for the inhibitory receptor named as CTLA-4, whose expression is induced on activated T cells [165]. Reduction in GVHD lethality was observed in mice treated with CTLA-4 Ig or infused with CD28-deficient donor T cells [166,167,168]. CTLA-4 Ig has been used as a surrogate ligand to block CD28/CTLA-4 T cell co-stimulation. CTLA-4 Ig treatment reduces the initial endogenous cytokine production and arrests the subsequent expansion of donor T cells, the differentiation of anti-host effectors, and the development of severe immune deficiency [167]. A limitation of this approach is that the blocking effect of CTLA-4 Ig is incomplete, presumably because of redundant roles of CD28 with other co-stimulatory molecules inhibition of Treg cell proliferation and the blockade of the negative signal transduction through CTLA-4, [165]. In another approach, lentiviral vector carrying CD28 shRNA was used to block the CD28/B7 signaling pathway, which allowed inhibition of T cell proliferation. This method reduced GVHD in a mouse model of allogeneic bone marrow transplantation [169].

#### 5.4.3. Inducible Co-Stimulator (ICOS)

ICOS is a member of the CD28 family that is expressed on activated T cells and memory T cells. It is constitutively expressed on B cells, macrophages, and dendritic cells and is upregulated on APCs and some non-lymphoid tissues by TNF or LPS [170]. Blockade or absence of ICOS diminishes GVHD [170,171]. Some studies have shown that inhibition of GVHD by ICOS blockade is associated with skewed differentiation towards Th2 cells [171]. However, other studies have showed evidence that blockade of ICOS results in lower proliferation of donor T cells and subsequent inhibition of GVHD [170].

#### 5.4.4. Programmed Death 1 (PD-1)

PD-1 is a member of the B7:CD28 superfamily and is involved in keeping the cellular immune system in check. PD-1 is expressed on activated CD4, CD8 T cells, as well as NK cells, B cells, and macrophages. PD-1 has two ligands, PDL-1 and PDL-2 that are expressed on APCs after cellular activation. Some studies have shown that blockade of the PD-1/PD-L1 pathway accelerates GVHD-induced lethality [172]. In accordance with these studies, studies have shown that GVHD is markedly accelerated in donor cells that lack PD-1 [80]. Inhibition of GVHD by PD-1/PD-L1 pathway correlates with the suppression of IFN-γ production [80]. Additional studies have shown that the PD-1/PD-L1 pathway is important to inhibit GVHD by Tregs. In this regard, the blockade of the PD-1/PD-L1 pathway abrogates the immunoregulation mediated by Tregs [173].

#### 5.4.5. The CD40/CD40-Ligand (CD40L) Pathway

CD40L pathway is involved in the GVHD lethality. CD40 is expressed on DCs and B cells, and CD40L is expressed on activated CD4^+^ T cells. CD40 signals activate DCs and activated DCs promote the activity of CD8^+^ T cells and CD4^+^ T cells. Studies using a DBA/2 (donor)→F1 (recipient) aGVHD or cGVHD model demonstrated that blockade of the CD40 pathway inhibits aGVHD and cGVHD [174]. Studies using conditioning GVHD models showed that CD40/CD40L interaction increases aGVHD lethality [175] by promoting direct CD4 T cell-mediated tissue damage and CD4^+^ T-cell proliferation [176]. Apart from the above-mentioned co-stimulatory molecules, inhibition of GVHD has also been possible by blocking members of the TNF superfamily of proteins including OX40, the herpes virus entry mediator (HVEM or TNFRSF14), and CD30. While the OX40/OX40L and CD30/CD30L pathways play a role in the CD4^+^ T cell-mediated GVHD [79], HVEM is involved in CD8^+^ T cell-mediated GVHD [177].

### 5.5. Molecular Targets in T Cells That Modulate Graft-versus-Host Responses

Recently, intracellular molecular targets in T cells have been recognized as being critical for regulating alloreactivity. Some of these intracellular targets include epigenetic regulation in donor T cells, notch signals in donor T cells, mitochondrial ATPase, the proteasome, protein kinases, microRNAs and the JAK-STAT pathways.

#### 5.5.1. Epigenetic Regulation in Donor T Cells

Epigenetic regulation in all cells is modulated via histone acetylation and methylation along with DNA methylation [178]. Histone deacetylase (HDAC) inhibitors are antitumor agents [179]. Some of these drugs have potent anti-inflammatory and immunomodulatory effects. This latter effect has shown therapeutic benefit after allo-HSCT in experimental models of GVHD. The regulation of HDAC by the HDAC inhibitors have been correlated in part with their ability to suppress the host antigen presenting cells such as DCs, Tregs and NK cells [179]. In a murine allogeneic bone marrow transplantation model, the pretreatment of DCs with HDAC inhibitors, such as suberoylanilide hydroxamic acid (SAHA) and ITF2357 reduced TLR-induced secretion of pro-inflammatory cytokines, reducing experimental GVHD [180]. Another study demonstrated that administration of DZnep, an inhibitor of histone methylation, arrested GVHD in mice after allo-HSCT. In this study, DZep promoted selective apoptosis in alloreactive effector T cells [181]. DNA hypo-methylating agents, such as decitabine (Dec) and azacitidine (AzaC), also reduced GVHD *in vivo* via a direct effects on donor Tregs [182].

#### 5.5.2. Targeting Notch Signals in Donor T Cells

Signaling mediated by Notch activation is important in cellular processes such as differentiation, survival and homeostasis [178]. In the setting of allo-HSCT Notch regulates hematopoietic progenitors and allo-immune T cells [183]. *In vivo*, studies have demonstrated that Notch regulates allo-reactive T cells that mediate GVHD. In mouse allo-HSCT models, inhibition of Notch in donor-derived T cells resulted in the reduction of severity and mortality of GVHD [183,184].

#### 5.5.3. Targeting Mitochondrial ATPase

During GVHD T cells proliferate in response to allo-antigens, and this process demands increased levels of ATP as a source of energy. Studies have revealed that proliferation of T cells increased aerobic glycolysis and oxidative phosphorylation. Alloreactive T cells also display increased superoxide production and decreased amounts of anti-oxidants. Bz423 is an inhibitor of the mitochondrial F_1_F_0_ adenosine triphosphate synthase (F_1_F_0_-ATPase), which induces selective apoptosis of alloreactive T cells, and reverses GVHD in BMT models without affecting hematopoietic engraftment or the immune reconstitution [185].

#### 5.5.4. Proteasome Inhibition

Proteasome inhibition has been shown to induce apoptosis of allo-reactive T cells [178]. Studies performed in mice have demonstrated that proteasome inhibitors such as bortezomib inhibits cytokine signaling and NF-κB activation [1]. Bortezomib preferentially and selectively depletes allo-reactive T-cells, supports Treg-cell survival, and attenuates IL-6 mediated T cell differentiation [186]. It also attenuates TLR4-mediated APC activation with reduced cytokine production and immunostimulatory activity. Significant protection from GVHD was observed when bortezomib was administrated to mice at the time of allo-BMT [186]. The effect of bortezomib on GVHD depends on the timing of its administration, as delayed administration causes exacerbation of GVHD in the gut, which is correlated with increased levels of type 1 interferon, TNF, interleukin-1beta (IL-1β) and IL-6 [187]. Thus, more effective methods that selectively deplete T cells from the donor graft may improve patient’s outcomes after allo-HSCT.

#### 5.5.5. Targeting Protein Kinases

Protein kinase Cθ (PKCθ) maintains the immunological synapse between T effector (Teff) cells and ligated-APCs, and is a key regulator of T-cell receptor (TCR) signaling. PKCθ promotes T-cell allo-reactivity and GVHD induction [188,189]. Studies using murine models of allo-HSCT have demonstrated that deletion of PKCθ leads to induction of T cell anergy [189] reducing the severity of GVHD [188]. Protein kinase Cα (PKCα) has recently been characterized as a cooperative and surrogate T cell activation signaling partner for PKCθ [190]. *In vivo* studies have demonstrated that both PKCα, and PKCθ contribute to GVHD. Pharmacological inhibition of PKCα and PKCθ with R524 prevented GVHD and preserved GVC responses. R524 hampers T cell proliferation *in vivo* and *in vitro*, and reduces the expression of chemokine receptors and the production of chemokines, preventing the development of GVHD [191].

#### 5.5.6. Therapeutic Intervention of GVHD by Targeting microRNAs

MicroRNAs are small single-stranded noncoding RNAs, which influence molecular pathways that control the development and function of innate and adaptive immune responses [192]. *In vivo* studies using mouse GVHD models showed that microRNA-155 (miR-155) was up-regulated during activation of T cells after allo-HSCT [193]. Lethal GVHD was markedly reduced in mice receiving miR-155 deficient donor T cells reduced compared with WT control mice [193]. Likewise, blocking miR-155 expression with a synthetic antibody after allo-HSCT decreased the severity of GVHD and prolonged survival in mice [193].

#### 5.5.7. Targeting Janus Kinase/Signal Transducer and Activator of Transcription Pathways

Regulation of donor T cells with specific signal transducer and activator of transcription (STAT) family proteins has shed light into the role of these proteins in GVHD regulation. Activation of STAT1 is an early event in GVHD and correlates with early cytokine storm [194]. Experimental evidence has revealed that lack of STAT1 in donor T cells reduced expansion of donor T cells and reduced GVHD [195]. Interestingly, deficiency of STAT1 inhibited the apoptosis of natural Tregs (nTregs), and facilitated the expansion of donor inducible Tregs (iTregs) [195]. STAT3 is another protein in GVHD-signaling pathway. In fact, activation of STAT3 correlates with the release of high levels of pro-inflammatory cytokines like IL-6 and also IL-10 [194]. In addition, studies have shown that elimination of STAT3 correlated with instability of nTregs and inhibition of iTreg cell polarization from naïve CD4^+^ T cells [196]. Transfer of STAT3-deficient naïve donor CD4^+^ T cells increased nTregs post-BMT and prevent GVHD lethality [196]. Murine model of allo-BMT showed that JAK3 is expressed in T cells and plays a role in the pathogenesis of GVHD [197]. Therefore, targeting JAK3 with the inhibitor JANEX-1 has prevented GVHD in mouse models [198]. Overall these studies suggest that targeting the JAK/STAT signaling pathway in donor T cells with small molecules could be effective for controlling GVHD.

### 5.6. Virotherapy as a Novel Strategy to Prevent or Control Graft-versus-Host Disease and Augment the Graft-versus-Cancer Effects after Allogeneic Hematopoietic Stem Cell Transplantation

Oncolytic viruses are promising new agents for targeting and killing cancer cells [199,200]. Among the rich diversity of candidate viruses that have been tested with different cancers, this review will focus on oncolytic MYXV. A prototypic member of the *Leporipoxvirus* genus of the *Poxviridae* family, MYXV causes the lethal disease called myxomatosis in European rabbits (*Oryctolagus cuniculus*) [201,202]. Interestingly, the virus is non-pathogenic for any host outside the lagomorph family, failing to propagate in any non-rabbit species, including immunodeficient mice and humans [201,203,204]. Despite its narrow host range in nature, MYXV productively infects and replicates in a wide diversity of human cancer cells. Two main factors have been characterized to date that render cancer cells more susceptible to MYXV infection. First, most cancer cells fail to mount a fully competent innate anti-viral response such as the synergistic effects of type I interferon and tumor necrosis factor pathways, which efficiently abort MYXV replication in normal primary human cells [205,206]. Second, many cancer cells have elevated levels of activated kinases such as Akt, which upregulate MYXV replication in cancer cells [207]. Thus, the induced activation of Akt using pharmacological manipulation can enhance MYXV replication in some refractory cancer cells, which are otherwise non-permissive [208]. The ability of MYXV to manipulate other intracellular anti-viral signaling pathways can also determine whether the virus will replicate in cancer cells, for example, via the inhibition of PKR signaling pathway. The MYXV dsRNA-binding protein M029 is essential for inhibition of PKR activation and replication in human cancer cells [209]. In another study it was also observed that MYXV preferentially infects cancer cells that possess dysfunctional or deleted p53, ATM and Rb tumor suppressor genes [207,210,211]. Studies evaluating MYXV have demonstrated the ability of this virus to target and kill many diverse human or murine cancers, including myeloid leukemia, multiple myeloma (MM) [5,8,212,213,214], melanoma, glioblastoma, ovarian cancer, and pancreatic cancer [214]. Importantly, these studies have demonstrated that MYXV selectively targets and kills malignant human hematopoietic cells such as leukemia cells, (MM), while sparing normal hematopoietic stem and progenitor cells (HSPCs) [8,213]. Therefore, MYXV is being developed as a novel *ex vivo* purging agent to functionally eliminate specific malignant cell populations from human hematopoietic stem cell transplants [8,9,212,213].

#### 5.6.1. *Ex Vivo* Treatment with MYXV Prevents GVHD in a Xeno-Transplanted NSG Murine Model

Recently, we reported that immunodeficient NSG mice xeno-transplanted with MYXV-pretreated human HSPC samples, such as BM or PBMCs, showed prolonged survival times compared to mock-treated transplant controls (e.g., without *ex vivo* MYXV treatment) [12]. In this model, sub-lethally irradiated adult NSG mice injected with either normal human BM or PBMCs displayed a lethal wasting disease by four to six weeks after transplant. The clinical presentation of this xeno-transplant-induced disease involved significant edema, and infiltration of CD3^+^ T cells into the liver, intestines, skin, lung, kidney and spleen, consistent with the classic pathology of allo-HSCT GVHD. On the other hand, mice injected with human BM or PBMCs that had been pretreated *ex vivo* with MYXV for 1 h prior to transplant survived until the time of sacrifice without evidence for the disease [12]. This study also demonstrated that the GVHD disease observed in the mock-treated xeno-transplanted recipients required mature human donor CD3^+^ T lymphocytes. In addition, *ex vivo* MYXV treatment of human HCT samples did not impair normal human hematopoietic stem and progenitor cell (HSPC) engraftment in the immunocompromised NSG recipients and yet selectively inhibited expansion of transferred donor CD3^+^ T cells in the transplant recipient tissues such as spleen, liver and lungs [12]. These results suggest that *ex vivo* pre-treatment of the human HCT specimens with MYXV attenuated the subsequent induction of GVHD. Importantly, the *ex vivo* MYXV treatment did not compromise the ability of the donor human HCT to mediate GVC as assessed by the elimination of pre-seeded human U266 MM in the majority of the recipient NSG mice.

In the same study, human-to-human haplo-mismatched mixed lymphocyte reactions (MLRs) were also tested to investigate whether MYXV could prevent the expansion of allo-reactive lymphocytes in the context of human antigen allo-stimulation. The results indicated that MYXV prevented responder cell proliferation in the MLRs, suggesting that MYXV prevents the expansion of alloreactive lymphocytes from primary human BM samples when stimulated with HLA-mismatched human donor leukocyte samples [12].

To summarize, two striking results were derived from this work [13]. First, *ex vivo* MYXV treatment for just 1 h prior to transplant affects the post-transplant activities of primary human CD3^+^ T lymphocytes found in HCT samples derived from either BM or PBMCs. Second, this *ex vivo* treatment of human HCT samples with MYXV delayed or eliminated the development of lethal GVHD in xeno-transplanted NSG mice, while sparing the GVC effects.

#### 5.6.2. MYXV Effects on Human T Cells

**Figure 2:**
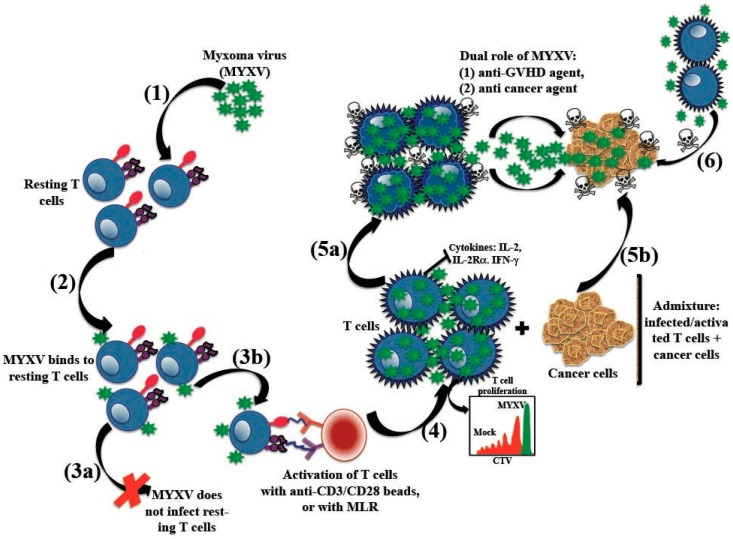
Dual effects of *ex vivo* virotherapy with myxoma virus: preventing graft-*versus*-host disease and yet preserving or augmenting graft-*versus*-cancer. Myxoma virus (MYXV) prevents graft-*versus*-host disease (GVHD) and improves the graft-*versus*-cancer (GVC) effects in six steps. (1) MYXV targets and (2) binds to resting human T cells in transplant samples. However, (3a) MYXV infection does not progress past the binding step in resting T cells unless (3b) T cells are stimulated (e.g., via anti-CD3/CD28 beads) or by allostimulation (e.g., via mixed lymphocyte reactions) when contacting allo-antigen in recipient tissues. (4) MYXV impairs T cell proliferation and secretion or endogenous production of inflammatory cytokines that fuel GVHD, including IL-2, IL-2R-α (CD25) and IFN-γ. (5a) MYXV reduces the viability of T cell inducing accelerated cell death. (5b) MYXV uses activated T cells as cell carriers to ferry virus to reach residual cancer cells in the transplant recipient. Once delivered, viral particles migrate from T cells to contacted cancer cells. This is followed by infection and killing of the target cancer cells. (6) In addition to this, activated T cells exposed to MYXV, but not productively infected, also become more efficient killers of cancer cells, suggesting that MYXV arms T cells to upregulate cytotoxic killing pathways against cancer cells, in addition to delivering oncolytic virus to the residual cancer.

Although *ex vivo* virotherapy with MYXV prevented GVHD caused by human T cells in the allo-HSCT xeno-transplant model [12], the mechanism by which the virus treatment prevented GVHD remained unexplained. However, recent *in vitro* studies performed with MYXV revealed that MYXV prevented GVHD by impairing the subsequent functionality of human T lymphocytes from the donor transplant [13]. This study demonstrated that, although MYXV binds to resting human CD3^+^ T cells, the activation of these virus-bound T cells is required to launch productive infection with MYXV. Importantly, the activation of MYXV-adsorbed human T cells caused the inhibition or suppression of T cell proliferation, as well as the downregulation of effector molecules such as IL-2, IL-2Rα and IFN-γ. These results help explain the mechanism by which *ex vivo* MYXV virotherapy prevents GVHD in the xenogeneic model [12]. Furthermore, these studies also demonstrated that MYXV can exploit activated human T cells as a cell carriers that can donate virus to infect and kill human MM cells (Figure 2 illustrates the proposed mechanism). Together, these *in vitro* studies suggest a dual role of *ex vivo* MYXV virotherapy to prevent GVHD, but yet also augment the GVC effects of donor human lymphocytes [13].

#### 5.6.3. MYXV Has a Dual Role as Anti-GVHD and Anti-Cancer Agent

Adoptive transfer of mature T cells contained in the allo-HSCT improves patient prognosis through the highly beneficial anti-tumor effects of GVC, which relies on donor T cells becoming engaged and activated in clearance of minimal residual disease [130]. This GVC effect, however, is eliminated upon complete depletion of donor T lymphocytes, a common strategy (whether chemically or physically) used to prevent GVHD. Since *ex vivo* MYXV virotherapy of human BM or PBMCs prevented GVHD in a xeno-transplanted model and yet poorly infected resting human T cells *ex vivo*, one intriguing question that needed to be addressed was how MYXV selectively prevented GVHD while still permitting GVC against pre-seeded human myeloma. Thus, by exploiting a xeno-transplant model that assessed both GVC and GVHD, we demonstrated that MYXV prevented GVHD did not impair either the GVC effects of the human HSPC transplant graft or the engraftment of the normal stem cells [12]. Importantly, the *ex vivo* pre-treatment of human BM with MYXV, followed by infusion in immuno-compromised mice with pre-seeded human myeloma, was a more effective anti-cancer strategy than just the direct injection of free MYXV virions, supporting the conclusion that the intravenous injection of free virus did not reach the pre-seeded disease in the BM as efficiently as when delivered via transplant leukocytes loaded *ex vivo* with virus [12]. Very recently, it was demonstrated that MYXV can exploit activated human T cells as cell carriers to deliver virus to human myeloma cells [13] (Figure 2). Interestingly, both input virus (“passive” mechanism) and progeny virus (“active” mechanism) from the infected T cells can be transferred to the target cancer cells. This infection leads to rapid cancer cell death, consisting with the oncolytic effects of MYXV infection on many human cancer cells (Figure 2). Interestingly, stimulated T cells that were not productively infected with MYXV in addition became better killers of cancer cells, suggesting that T cells exposed to MYXV are also better armed to kill cancer cells by direct cytolysis. These latter results indicate that MYXV enhances the beneficial effects of GVC by multiple mechanisms [13]. Thus far, MYXV is the only live oncolytic virus that has been exploited for the prevention of GVHD.

## 6. Conclusions

Despite significant progress in allo-HSCT, GVHD remains a major clinical complication of this therapy. Many therapeutic strategies have emerged in the last two decades with the aims of preventing or controlling GVHD and improving patient outcomes after allo-HSCT. Unfortunately, most strategies also reduce GVC against residual disease remaining in the patient and have only shown incremental benefits in mitigating GVHD. Development of safer strategies to prevent and treat GVHD, while still preserving or even augmenting GVC, will particularly help higher-risk transplant populations. Populations at higher risk for GVHD include those who are older or lack a suitably matched donor, including racial and ethnic minorities. *Ex vivo* oncolytic virotherapy with MYXV may be a promising strategy for preventing GVHD while simultaneously augmenting GVC. In this regard, understanding the molecular mechanism(s) by which MYXV mediates these dual effects will be critical to translate this new technology to the clinic.

## Figures and Tables

**Table 1:** Mediators of acute graft-*versus*-host disease.

Mediators of aGVHD	Examples	Functions in the Context of aGVHD	Ref.
**T cell co-receptors for MHC class I and class II**	CD4^+^ (MHC-class II) and CD8^+^ (MHC class I) T cells.	- In the majority of HLA matched HCT, CD4+ or CD8+ or both are induced in response to mHAs.	[81]
**Naïve and memory T cells**	CD62L^+^ CD44^−^ (naïve T cells); CD62L^+^ CD44^+^ (central memory T cells); CD62L^−^ CD44^−^ (terminally differentiated effector/effector memory).	- Donor naïve CD62L^+^ are the primary alloreactive cells that enhance the GVHD - Induction of anergy of alloreactive naïve T cells is possible with co-stimulation blockade, deletion via cytokine modulation among others. - While memory and alloreactive T cells can cause severe GVHD, memory T cells that are not alloreactive do not cause GVHD, yet transfer functional memory that mediates GVL effects	[82,83,84,85,86]
**Th1 subset**	IFN-γ, IL-2 and TNF-α.	- IL-2 production by donor T cells is the main target of prophylactic approaches and treatment of GVHD. - The role of Th1 cytokines is complex. In fact, they can be regulators or inducers of GVHD.	[87,88]
**Th2 subset**	IL-4, and IL-10, G-CSF, IL-4 and IL-18.	- Lack of secretion of Th2 cytokines by donor T cells is reflected in an increase in the severity of GVHD. - Some studies have demonstrated that polarization of donor T cells toward the production of Th2 cytokines reduces Th1 production and the severity of aGVHD. However, other studies have failed to demonstrate the beneficial effect of Th2 polarization in aGVHD - IL-18 pre-treatment has been associated with reduced IFN-γ and higher IL-4 secretion.	[34,59,89,90,91]
**Th17 subset**	IL-17	- The role of IL17 in GVHD remains unclear. In fact, some studies have shown IL-17 contributes to the early development of GVHD by promoting the induction of inflammatory cytokines. In contrast, other studies have shown that absence of IL-17 in donor T cells increases Th1 differentiation, IFN-γ production and exacerbates aGVHD in allogeneic recipients.	[34,92,93]
**Regulatory T cells (Tregs)**	CD4^+^CD25^+^Foxp3^+^ (nTregs)	- nTregs suppress the proliferation of effector allo-reactive donor T cells resulting in the prevention aGVHD. - Also, after allo-HSCT viral immunity is preserved in the presence of Tregs. - However, Tregs can prevent GVL depending on the ratio of effector T cells to Tregs.	[94,95,96]

GVHD: graft-*versus*-host disease; aGVHD: acute graft-*versus*-host disease; allo-HSCT: allogeneic hematopoietic stem cell transplantation; G-CSF: granulocyte colony-stimulating factor; GVL: graft-*versus*-leukemia; IFN: interferon; HCT: hematopoietic cell transplant; HLA: human leukocyte antigen; MHC: major histocompatibility complex; mHa: minor histocompatibility antigen; IL: interleukin; Ref: reference; nTregs: naturally occurring Tregs.
